# Management peculiarities of costovertebral hydatidosis

**DOI:** 10.4314/ahs.v22i2.26

**Published:** 2022-06

**Authors:** Sarra Zairi, Amany Touil, Taher Mestiri, Khaled Hadhri, Mondher Mestiri, Adel Marghli

**Affiliations:** 1 Department of thoracic surgery. AbderrahmaneMami Hospital - Ariana. Medical school of Tunis – University of Tunis El Manar, Tunisia; 2 Department of anesthesiology. AbderrahmaneMami Hospital - Ariana. Medical school of Tunis – University of Tunis El Manar, Tunisia; 3 Department of Orthopedics and Traumatology. Charles Nicolle Hospital. Medical school of Tunis – University of Tunis El Manar, Tunisia; 4 Department of Orthopedics and Traumatology. Kassab Institute. Medical school of Tunis – University of Tunis El Manar, Tunisia

**Keywords:** Costovertebral region, spinal cord compression, echinococcosis

## Abstract

**Background:**

Costovertebral hydatidosis is a rarely reported clinical and radiological entity, estimated at less than 1% of thoracic hydatid locations. Its management is still not codified.

**Objective:**

The aim of our study was to specify the management peculiarities of costovertebral hydatidosis.

**Methods:**

Between January 2000 and December 2018, 14 patients were managed for costovertebral hydatidosis in a thoracic surgery department.

**Results:**

The mean age of our patients was 48 years. The history of a prior hydatid disease was found in 7 patients. Imaging features were suggestive in 13 cases. They showed: involvement of the spinal canal (6 cases), of the soft tissues (5 cases) and spinal cord compression (3 cases). Costovertebral resection of the hydatid lesions was complete in 12 cases. Four patients presented postoperative complications.

**Conclusion:**

Costovertebral hydatid involvement, may threaten the functional and vital prognosis. Therefore, early diagnosis and management are mandatory, before the occurrence of irreversible neurological impairment. Surgical resection remains the treatment of choice and must be complete whenever possible. Relapse is frequent, hence the importance of a regular follow-up.

## Introduction

Hydatid disease is a cosmopolitan zoonosis caused by the development in humans of a taenia: Echinococcusgranulosus, in its larval form.^1^ It is endemic in several countries, with traditional pastoral farming practices. Hydatid cyst (HC) is more frequently located in the liver and lung. Bone involvement remains rare (2%). Vertebrae and ribs are involved in 35 and 6% of the cases; respectively.^2^ Costovertebral hydatidosis (CVH) is a specific clinical and radiological entity, which requires particular surgical management. Although benign, it may threaten the functional and vital prognosis. Because of its rarity, few studies have been interested in this location and its management remains unclear.^3^,^4^ We reported herein, our experience in the management of CVH, through which we studied the peculiarities of this location.

## Methods

**Figure 1:**
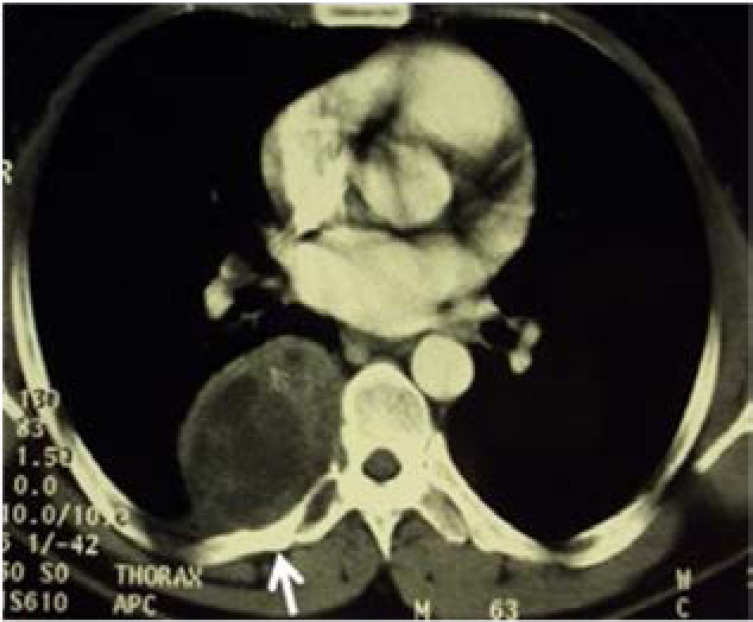
Transverse section thoracic CT, showing a multivesicular fluid lytic mass involving the transverse process and pedicle of the 7th dorsal vertebra and the posterior arch of the 7th rib (arrow).

Between January 2000 and December 2018, 14 patients were managed for a CVH in our thoracic and cardiovascular surgery department. The study was approved by the local hospital ethics committee. Data were retrospectively gathered from the patient's medical records and analyzed in terms of symptoms, radiological findings and surgical techniques and outcomes. All of our patients had complete physical examination and chest radiography. Chest and abdominal computed tomography (CT) scans were performed in all cases. They allowed assessment of bone involvement and extent of hydatic disease to the neighbouring soft tissues, lung parenchyma, pleura and spinal canal (figure 1).

**Figure 2:**
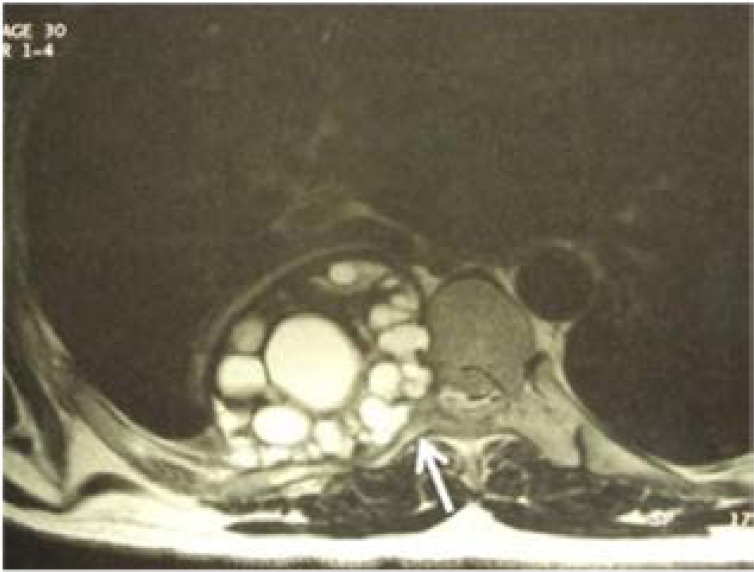
Transverse section thoracic MRI showing right multivesicular lesion of the costovertebral gutter, with involvement of the posterior arch of the 7th rib and the 7th dorsal vertebra (arrow), presenting a foraminal and endocanalar extension.

T1 and T2-weighted magnetic resonance imaging (MRI) were performed, to further study bone, soft tissue, spinal canal and spinal cord involvement, in 10 cases (figure 2).

Other tests were carried out inconstantly: thoracic ultrasound and hydatid serology.

Patients were managed by a multi-disciplinary surgical team of thoracic surgeons and orthopedists. Surgical approach was selected according to the cyst's location, size and number, the extent of the lesions to the spinal canal, vertebrae and neighboring structures. Posterolateral thoracotomy directly above the affected rib was performed in all cases, associated with a posterior vertebral approach in 2 cases.

Protection of the operative field with hypertonic saline soaked gauzes was systematic. Abundant washing of the operative field with hydrogen peroxide or povidone iodine were systematic, in order to avoid the spread of the parasite and secondary hydatic lesions.

**Figure 3:**
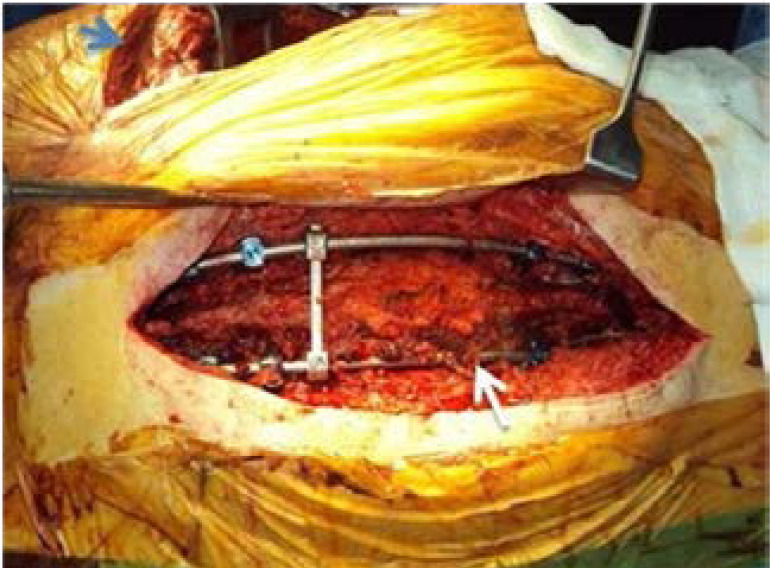
First posterior spinal fixation (white arrow) before the anterior thoracic time for hemi-vertebrectomy in a patient previously operated for CVH. Note the double approach, with posterolateral thoracotomy (blue arrow).

Associated neighbouring locations, were treated at the same time. Pulmonary or pleural cysts were treated at first, then the CVH. Treatment of the HC included two steps. The first one consisted in treatment of the parasite. After sterilization with hypertonic saline solution and suction of the cyst's content, the germinative membrane was removed. Treatment of the residual cavity was performed second. For the associated locations, it was conservative and consisted in pericystectomy. For the bone lesions, it was radical. One to 3 ribs were removed on average. Depending on the extent of vertebral involvement, corporectomy, either total or partial, was performed in 7 cases. Between 2 and 4 vertebrae were resected and spinal osteosynthesis was performed in 5 patients with bone grafts (figure 3).

Medical treatment with Albendazole (10 mg/kg/day) was systematically prescribed preoperatively, at the time of imaging diagnosis. According to the completeness of surgery and CT features of cyst's viability at follow-up, courses of Albendazole were maintained postoperatively, from 6 to 18 months, with a daily intake for 21 days and an interval of 7 days between courses.

## Results

Fourteen patients were included in the study, with a sex ratio of 1.8 (9 men and 5 women). The mean age was 48±15.4 years [27–76 years]. History of a previously operated HC was found in 7 patients and 4 among them were in the costovertebral setting (table 1).

**Table 1:** History of previous hydatid disease operated in our series

Characteristics Patient	Number of interventions	Hydatic location
1	1	liver
2	1	lung
3	3	thoracic soft tissues
4	Multiple	multiple (liver, left kidney, lung, pleura, ribs and vertebrae)
5	7	secondary pleural and costovertebral with invasion of the spinal cord
6	1	costovertebral with spinal cord compression
7	1	complex vertebral with paraplegia

Clinical symptoms were dominated by back pain (7 patients) and neurological signs (4 patients), such as: paraparesis (2 patients), paraplegia with sphincter disorders (1 patient) and paresthesia of the two lower limbs with a decreased functional walking distance (1 patient). Frank hemoptysis was reported in 2 cases. Four patients reported the history of a several-year-evolving paravertbral swelling, which became recently painful. Discovery was fortuitous in one case, at a thoracic CT, performed during follow-up for a previously operated soft-tissue HC. Imaging investigations, were suggestive of CVH in 13 cases and the diagnosis of a chondro-sarcoma was evoked in one case. Hydatic lesions were right sided in 11 cases (table 2).

**Table 2:** Imaging findings

Imaging	Findings	Number
Chest radiography	Water tone opacity	14
Chest ultrasound	Compressive multivesicular parietal cystic formation	5
	“double line sign”	1
Chest and abdominal CT	Right side	11
	Concomitant hydatid location:	
	- pleura	2
	- lung	1
	- liver	1
	Rib involvement	
	- 8th	9
	- 7th	7
	- 6th	5
	- 9th	4
	Multiple	9
	Bone destruction	7
	- Cortical rupture	2
	Soft tissues involvement	5
	Spinal canal involvement	6
MRI	Spinal canal involvement (unseen in CT)	2
	Spinal cord compression	3

Assessment of hydatic lesion extent showed that, the 8th rib was the most frequently involved (9 cases). Multi-level involvement was seen in 9 patients. Involvement of the spinal canal was shown in 6 patients and spinal cord compression in 3 cases.

Surgical resection was performed in all cases. Decompression laminectomy was necessary and performed at first in 2 cases, in the neurosurgery department. Complete resection of all the hydatid lesions was achieved in 12 cases (table 3).

**Table 3:** Performed procedures

Procedures	Number
Rib head disarticulation	14
Transversectomy	14
Removal of ribs (1 to 3)	14
Corporectomy	7
-total	3
-partial	4
Incision and drainage of an infected cyst	1
Removal of hydatid material from the spinal canal	3
Spinal osteosynthesis	5
Bone graft	5
-iliac	2
-fibular	2
-costal	1
Removal of a soft tissue HC	1
Pleurectomy for a secondary pleural hydatidosis	1
Pericystectomy for a lung HC	1

Medical treatment with antibiotics was necessary in 1 case pre-operatively, due to drainage of an infected HC to the skin. The mean hospital stay was 9.4±8 days [4–33 days]. The postoperative course was complicated in 4 cases: pulmonary embolism associated with flaccid paraplegia related to spinal cord ischemia in 1 case, spastic paraplegia due to a non-compressive spinal hematoma, which regressed spontaneously in 1 case and post-operative fever in 1 case. One patient presented a postoperative pneumonia and died 12 days after the operation, secondary to acute respiratory distress syndrome. Recovery from neurological impairment was complete in 3 patients. During follow-up, only one patient previously operated for pleural and costovertebral hydatidosis, presented a relapse after 8 years, with progressive onset paraplegia and anal incontinence secondary to hydatid spinal cord compression, requiring emergent surgery for decompression and reoperation. There were no side effects reported secondary to Albendazole treatment.

## Discussion

We reported our experience about 14 patients, managed for a hydatid disease in a rather rare setting. Costovertebral location is rare, estimated at less than 1% of thoracic hydatid locations.^5^ In a report about 1619 cases managed for thoracic hydatidosis, 8 patients only were operated for costal hydatidosis.^5^ Costal and vertebral involvement could be either primary, through the blood stream, or secondary, to an intra-thoracic location with intra-operative local parasite seeding. Because of the resistance of the bony tissues, the growth of the cyst is rather slow and the patient can remain asymptomatic for a long time, up to 10 to 20 years.^1^,^6^,^7^ The developing hydatid larva, increasing in size and volume, is put under pressure and intra-cystic multi-vesiculation occurs, as a form of suffering of the cyst. In the long run, surrounding bone tissues compression, results in cortical destruction, causing parasite spreading to the neighbouring structures.^1^ Main reported chief complaints are chest pain, parietal swelling or a pathological fracture.^1^,^5^,^8^–^10^ Half of our patients presented for back pain and parietal swelling was reported in 4 cases. Involvement of the spinal cord is announced by neurological signs such as: gait disturbances, spinal claudication or sphincter disorders.^8^–^12^ The risk of paraplegia or spinal cord compression has been estimated about 25 to 80% of patients with vertebral hydatid damage.^13^ In our series, neurological signs were reported in 4 cases: paraplegia with sphincter disorders in 1 case, gait disturbance in 1 case and paraparesis in 2 cases. Imaging confirmed the spinal cord compression in 3 cases. Imaging investigations are paramount in the diagnosis and assessment of CVH lesions. Chest x-ray typically shows confluent lacunae images with blurred boundaries, of variable size, without periosteal reaction, or alteration of the bone general morphology. The lesion often involves the posterior arch of the rib and extension to the adjacent vertebrae is of varying severity.^4^ Thoracic ultrasound is very useful for the diagnosis and assessment of HC of the chest wall, with diaphragmatic or pleural involvement.^14^ But, in case of costovertebral extension, CT-scan allows assessment of vertebral and possibly spinal canal involvement.^4^ Spinal canal involvement, spinal cord compression and soft tissues involvement are better assessed with MRI.^4^,^15^ In our series, MRI showed spinal canal involvement misdiagnosed at CT in 2 cases, spinal cord compression in 3 cases and better assessed neighboring soft tissues extension in 5 cases. Serological tests help confirm the parasitic origin and eliminate differential diagnoses. Their positivity is proportional to the cyst's size and integrity.^1^ However, serology can be negative in case of aged, calcified or involuted HC. CVH management is essentially based on surgery.^5^,^14^ The surgical approach depends on the extent of the lesions and the operative strategy. Postero-lateral thoracotomy allows extended costal resections of the chest wall, access to all the thoracic vertebrae, with the possibility of reconstruction and osteosynthesis through a combined posterior vertebral approach. Resection of an associated mediastinal or pulmonary location is also feasible.^12^,^16^ The operative sequence is determined by the planned procedures. Surgical resection should be complete and broad, whenever possible, removing all the hydatid cysts without rupture, the osseous and extraosseous lesions. Spinal cord injuries are common in CVH.^12^ In our series, spinal cord compression was observed in 3 cases and spinal canal involvement in 6 cases. In case of spinal cord compression signs, surgical decompression is urgently required. Laminectomy and transverse processes or vertebral pedicles resection, followed by bone graft and osteosynthesis, are then performed.^9^ Two patients required laminectomy at first in our series. Due to the slow development of HC, recovery from neurological injuries is possible, provided that surgical resection is complete and early.^9^,^10^,^12^,^17^ This was the case in our patients. Prolonged medical treatment with Albendazole is recommended perioperatively, in order to avoid relapses and whenever resection is not feasible or incomplete.^17^,^18^

A previous study, had shown that Albendazole should be given, for at least 6 months, so that the levels of circulating antigen, indicating the viability and biological activity of the parasite, become negative.^19^

In our series, medical treatment with Albendazole (10 mg/kg/day) was systematically prescribed preoperatively. According to the completeness of surgery and CT features of cyst's viability at follow-up, courses of Albendazole were maintained postoperatively, from 6 to 18 months, with a daily intake for 21 days and an interval of 7 days between courses.

In the same study,^19^ Albendazole, was rather well tolerated, and there were no reported side effects. This was the case in our series too.

Risk of recurrence and death was high in the past, due to delayed diagnosis, incomplete resection and different surgical techniques, including an exclusive posterior approach. 20,21 However, a review of recently published papers has shown an improvement in prognosis.^3^,^4^,^10^,^12^ A prolonged and regular follow-up, based on clinical examination, serology and CT-imaging is mandatory, in order to watch for the slightest recurrence. In our series, 4 patients had already been operated before and one patient presented a recurrence 8 years later. Management of recurrences is based on surgery as much as possible, except in cases where the vertebral involvement is complex and complete resection of the lesions is no longer feasible, due to their extent or due to prior resections.

## Conclusion

CVH is difficult to diagnose, assess and manage. Spinal cord involvement is the main prognostic factor. Therefore, MRI should be systematically performed, in order to diagnose and assess spinal canal involvement as early as possible. Surgical resection remains the treatment of choice and must be complete whenever possible. Relapses are frequent and anti-helminthic treatment is paramount postoperatively. Prolonged and regular follow-up and preventive measures are necessary to avoid parasitic reinfection.

Presented as: a poster at The 23rd French-language pulmonology congress in Marseille, France in January 2019, with a published abstract in French in Revue des Maladies Respiratoires, Volume 36, Supplement, January 2019, Page a221.
